# Dark States in the Light-Harvesting complex 2 Revealed by Two-dimensional Electronic Spectroscopy

**DOI:** 10.1038/srep20834

**Published:** 2016-02-09

**Authors:** Marco Ferretti, Ruud Hendrikx, Elisabet Romero, June Southall, Richard J. Cogdell, Vladimir I. Novoderezhkin, Gregory D. Scholes, Rienk van Grondelle

**Affiliations:** 1 Department of Physics and Astronomy, VU University, 1081 HV Amsterdam, The Netherlands; 2 Division of Biochemistry and Molecular Biology, Institute of Biomedical and Life Sciences, University of Glasgow, Glasgow G12 8QQ, UK; 3 A. N. Belozersky Institute of Physico-Chemical Biology, Moscow State University, 119991 Moscow, Russia; 4 Department of Chemistry, Princeton University, Washington Rd, Princeton NJ 08544, USA

## Abstract

Energy transfer and trapping in the light harvesting antennae of purple photosynthetic bacteria is an ultrafast process, which occurs with a quantum efficiency close to unity. However the mechanisms behind this process have not yet been fully understood. Recently it was proposed that low-lying energy dark states, such as charge transfer states and polaron pairs, play an important role in the dynamics and directionality of energy transfer. However, it is difficult to directly detect those states because of their small transition dipole moment and overlap with the B850/B870 exciton bands. Here we present a new experimental approach, which combines the selectivity of two-dimensional electronic spectroscopy with the availability of genetically modified light harvesting complexes, to reveal the presence of those dark states in both the genetically modified and the wild-type light harvesting 2 complexes of *Rhodopseudomonas palustris*. We suggest that Nature has used the unavoidable charge transfer processes that occur when LH pigments are concentrated to enhance and direct the flow of energy.

Light harvesting is one of the most efficient processes in photosynthesis, with a quantum efficiency approaching unity in spite of the highly energetically disordered nature of the pigments and proteins involved. In purple photosynthetic bacteria this is accomplished by specialized membrane-bound pigment-protein complexes, so-called light-harvesting or LH complexes, which form networks of coupled pigment molecules (chromophores)^1^,^2^,^3^. After the solar energy is captured by the chromophores, the electronic excitation is transferred within networks of light-harvesting molecules and funnelled to energy traps (reaction centres) on a 10–100 ps timescale^1^,^2^. The presence of both pigment-pigment and pigment-protein interactions produces a highly congested energy landscape, with the presence of excitonic, vibronic and charge-transfer states^3^,^4^,^5^,^6^.

Energy transfer in photosynthetic complexes has been the subject of detailed studies during the past few decades^1^,^7^,^8^,^9^,^10^,^11^, and recently it has been proposed that the coupling between vibronic and charge transfer (CT) states promotes the high efficiency of the first steps of charge separation in the PSII reaction centre at physiological temperatures^12^,^13^. Considering that evidence suggests that CT states are also present in strongly coupled complexes such as LH1^11^,^14^ and LH2^4^ from purple bacteria, we set out to try to detect low-lying dark states, possibly CT states, in LH2. We used two-dimensional electronic spectroscopy (2DES) to compare two types of LH2 complexes isolated from *Rhodopseudomonas palustris*. 2DES is particularly useful to investigate energy transfer in multi-chromophoric systems, especially if it involves almost dark states such as CT states, because 2DES decongests overlapping transient spectra as has been shown for dark states of carotenoids^15^. The 2DES technique has earlier been used to study the energy transfer and the electronic structure of LH2^16^,^17^,^18^ and in a variety of other photosynthetic complexes^12^,^19^,^20^,^21^. By comparing the two forms of LH2 using 2DES we have discovered a low energy dark state that may play a crucial role in the efficiency of light harvesting.

LH2 is a peripheral antenna that absorbs solar light and delivers the electronic energy to the core light harvesting complex LH1 and, in turn, to the reaction centre where photo-excitation drives a trans-membrane charge separation. LH2 has a ring structure, typically comprising 9 αβ protein subunits. Each αβ subunit binds 3 bacteriochlorophylls (BChl), that are arranged in two C_9_-symmetry rings (Fig. 1b). One ring consists of weakly coupled BChl ‘monomers’, absorbing at 800 nm (constituting the B800 band), whereas the other ring contains strongly coupled BChl dimers, which typically have an absorption peak at 850 nm (the B850 band). This is the case for the LH2 complex isolated from *Rps. palustris* grown under high light conditions (wild type or WT-LH2)^22^. This is illustrated in Fig. 1a in red where the two separate absorption bands can be clearly seen. However, there is no high-resolution crystal structure available for any LH2 complex from *Rps. palustris*, but C_9_-symmetry best describes data obtained from low resolution crystal data and from single molecule spectroscopy experiments^22^, which strongly suggest the presence of 9 αβ subunits. LH2 complexes from *Rps. palustris* typically have a heterogeneous polypeptide composition; in fact wild type cells have a multi-gene family of *puc* genes that encode the LH2 apoproteins. Interestingly it has been possible to obtain a deletion mutant of *Rps. palustris* that only has one pair of the *puc* genes left: the *puc*D αβ genes (Southall and Cogdell, unpublished data). This mutant only expresses an LH2 complex when it is grown under low light conditions, and the particularity of this LH2 complex (M-LH2) is that the B850 band is blue-shifted by about 40 nm and therefore merges with the B800 band, resulting in a quasi-single absorption peak in the 800 nm spectral region (the B810 band), as shown in Fig. 1a in blue.

## Results

### Two-dimensional electronic spectra

A 2DES experiment records 2D spectra, which correlate absorbed frequencies *ω*_*τ*_ with radiated frequencies *ω*_*t*_ of a sample for each population time (*T*). In the 2DES experiment three ultra-short and spectrally broad laser pulses, delayed in a controlled manner, excite and probe the sample. After this sequence of pulses, the stimulated photon echo is emitted by the complexes and is recorded in the frequency domain *ω*_*t*_ as a function of coherence times *τ* (time difference between the first and second pulse) and population time *T* (time difference between the second and third pulse). Fourier transformation with respect to *τ*, yields the 2D spectra as a function of excitation frequencies *ω*_*τ*_ for each time *T*. The *T* evolution of the 2D spectra contains information about energy transfer in the amplitude of non-oscillating cross-peaks^12^,^23^ and it is sensitive to the presence of vibronic /excitonic coherences as amplitude oscillations in both diagonal and off-diagonal peaks^12^,^20^,^24^,^25^,^26^,^27^,^28^.

**Figure 2:**
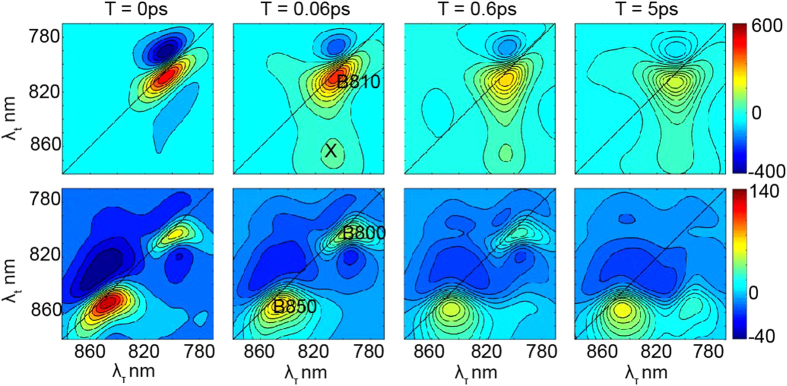
2DES real total spectra. Real total (rephasing plus non-rephasing) 2D spectra at different population times *T* = 0 ps, 0.06 ps, 0.6 ps and 5 ps for M-LH2 (top) and WT-LH2 (bottom). The colour represents the intensity of the 2DES spectra in arbitrary units since it is the results of a fast fourier transformation. Opposite to a transient absorption experiment, the intensity corresponding to ground state bleaching and the stimulated emission is positive, whereas the intensity corresponding to excited state absorption is negative. M-LH2 shows a positive peak along the diagonal emitting at 810 nm (B810 GSB and SE), a negative above diagonal peak emitting at 790 nm (B810 ESA) and a positive below diagonal peak emitting at 850nm (positive, X state). The WT-LH2 shows two positive diagonal peaks emitting at 805 and 850 nm (B800 and B850 GSB and SE), a negative above diagonal peak emitting at 830 nm (B850 ESA) and a positive below diagonal peak emitting at 850 nm.

**Figure 3:**
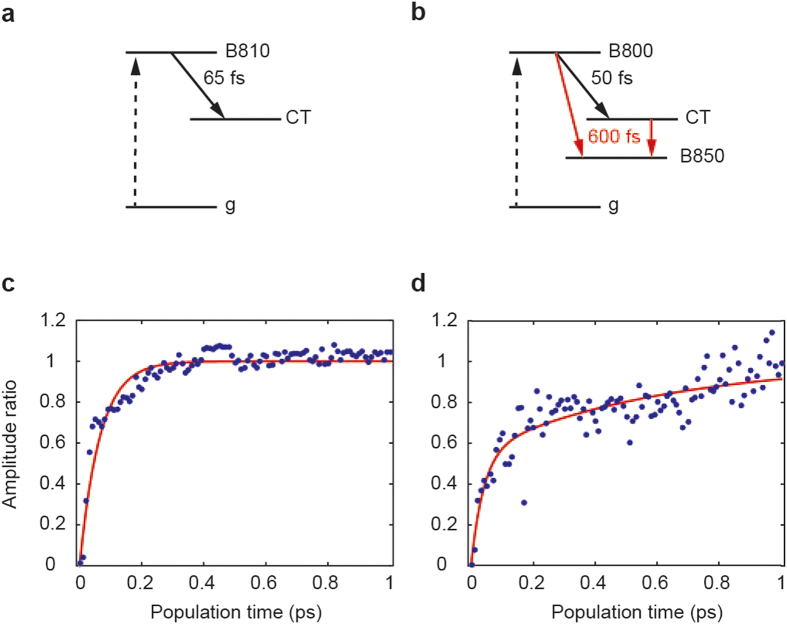
Energy transfer. (**a**) Energy level scheme for M-LH2. The dashed arrow represents the excitation due to absorption of light and the solid arrow represents excitation energy transfer or EET. (**b**) Energy level scheme for WT-LH2. (**c**) Amplitude of the positive below diagonal peak (810, 850) nm, divided by the diagonal peak (810, 810) nm at each respective time point, for M-LH2 (dots). The line corresponds to the exponential fit (one component). (**d**) Amplitude of the positive below diagonal peak (805, 850) nm, divided by the diagonal peak (850, 850) nm for WT-LH2 (dots). The line corresponds to the two-exponential fit.

We measured the 2D spectra for two forms of detergent-isolated LH2 complexes suspended in buffer solution, one isolated from wild type LH2 grown under high light intensity, WT-LH2, and the other from the *puc*D only deletion mutant grown under low light, M-LH2. The 2D spectra at some representative population times are shown in Fig. 2. The elongated features on the diagonal represent the decay of the bleach of the exciton states^12^. In the WT-LH2 spectra (Fig. 2, bottom) the bleach of the B800 and the B850 bands appears at 805 nm and 850 nm, respectively. In contrast, the M-LH2 spectra (Fig. 2, top) have only one peak at 810 nm due to the overlap between the B800 and the B810 bands, resulting in a single peak in absorption: the B810 band. In the latter, at zero population time (*T* = 0 ps), there are two negative cross-peaks, which represent excited state absorption (ESA) of the excited states producing bleaching at B810 nm. After 0.06 ps a positive cross-peak appears at (*ω*_*τ*_,*ω*_*t*_) = (810,850) nm, and the intensity grows with time *T* as shown in Fig. 3c. This reveals population transfer from the exciton state at 810 nm (the B810 band) to an almost-dark state X, detected at 850 nm. The absence of this peak at time zero shows that the X state is mostly populated by excited state transfer from states absorbing at 810nm (B810) and not by direct light excitation. This hypothesis is also confirmed by the low intensity of the corresponding diagonal peak at 850 nm. Notice that in Fig. 3b, the cross-peak amplitude has been normalized to the amplitude of the diagonal peak at (810,810) nm. The normalization is applied to compensate for the fast component decay (less than 50 fs) of the 2DES amplitude, which is due to dephasing of the initial exciton coherence created by the excitation pulses; in fact this dephasing occurs in both diagonal and cross-peaks.

In the WT-LH2 2D spectra, at *T* = 0 there are 3 negative off-diagonal peaks corresponding to ESA of the states absorbing at 800 nm (B800) and at 850 nm (B850). However, in this case the positive off-diagonal peak at (805,850) nm is already present at time zero. This peak originates from the excitonic coupling between the B800 and the B850 bands. The time evolution of the cross-peak intensity, normalized to the diagonal peak intensity at (850,850) nm, grows with time *T* (Fig. 3c). This implies that also in this case, like in the M-LH2, there is a signature of energy transfer from the states absorbing at 800 nm (B800) to a state emitting at 850 nm. Normally it is considered that growth of this amplitude is caused by B800 to B850 energy transfer. However, analysis of this kinetics (see next section) shows the presence of a bi-exponential rise, suggesting the presence of the X state in the WT-LH2 as well.

Over the past decades the B800 and the B850 exciton manifolds have been the objects of intensive theoretical studies^3^,^4^,^29^, and it has been shown that both static and dynamic disorder values for the B850 band are 4–5 times bigger than those of the B800 band. This difference has been explained by the formation of CT states in the B850 band^4^, due to the presence of closely spaced pigments in the B850 ring. The CT states are localized electron hole pairs, which have a static dipole moment different from zero and a transition dipole moment equal to zero. However, when CT states are mixed with exciton states, their transition dipole moments will be non-zero, and these states can be directly excited from the ground state. This mixing leads to excitons that exhibit a non-uniform electron density, providing ultrafast channels for energy transfer and charge transfer/separation^12^,^30^. Evidence for the presence of CT states in LH2 complexes is seen in Stark spectroscopy measurements^14^,^31^, where it has been shown that CT states, mixed with the lowest exciton state, are required in order to explain the high values of the Stark parameters (difference dipole moment **Δμ** and difference polarizability **Δα**). Other evidence for CT states in LH2 has been provided by ultrafast fluorescence measurements^29^, where CT-exciton interaction is needed to explain the interband energy transfer rate.

The dark X state emitting at 850 nm in M-LH2 has properties consistent with these previous studies and, therefore, we propose that this state is the low lying CT state of LH2. However, it is important to mention that as it has been shown by theoretical models^32^ that low lying CT states are highly unusual in nanoscale systems, such as molecular aggregates. In fact, polaron pairs also play a role in the dynamics of photosynthetic complexes and in molecular aggregates. A polaron represents a lattice distortion, and has many similarities with CT states^33^. Evidences for the presence of polaron pairs in LH2 complexes are provided by analysis of ultrafast transient absorption studies, however it is important to notice that in this work there is also strong evidence for low lying CT states in LH2^34^. It is, therefore, not possible to fully understand whether the observed X state is a CT state or a polaron or a combination, without further theoretical modeling of the data.

### Time evolution of positive cross-diagonal peaks

The time-evolutions of the positive off-diagonal peaks (805,850) nm of M-LH2 and WT-LH2, normalized to the corresponding diagonal peak intensity, are shown in Fig. 3c,d. In both cases, after normalization, the traces show that the amplitude grows to a maximum level, which corresponds to depletion of the initially populated B800 or B810 states, due to energy transfer to the X or to the B850 states. The main difference is that in the former the intensity saturates faster (in less than 100 fs) than in the latter.

In order to measure the time constants, an exponential fit has been performed, using the following model:


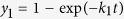



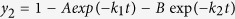


where *y*_*1*_ and *y*_*2*_ are the normalized amplitudes, respectively for M-LH2 and WT-LH2, *k*_*1*_ is the fast rate and *k*_*2*_ is the slow rate. The result is one component of 65 fs for the M-LH2 and two components of 50 fs and 600 fs for the WT-LH2. In the latter case the presence of two time constants is in agreement with what was earlier found in the anisotropy decay of transient absorption of other LH2 complexes^29^ and in previous 2DES measurements^17^.

Whereas it is clear that in M-LH2 there is an X state emitting at 850 nm, which is populated in 65 fs, the evidence for the presence of an X state in WT-LH2 is less direct. But the presence of a fast component of 50 fs suggest the presence of the X state, since the fast phase is similar to the one seen with the M-LH2. However, in WT-LH2 it is not possible to locate its exact position, because of the presence of the B850 exciton band, which overlaps with the X state. In addition, in case in the WT-LH2 when a CT state is present and quasi-degenerate with the major excitonic transitions they will become strongly mixed and maybe not easily distinguishable (see below). It is important to note also that the pigment composition of the M-LH2 complex and its CD spectrum demonstrate that it has the same overall standard LH2 structure as the WT-LH2 complex but the binding sites of the strongly coupled Bchls have changed so that the B850 band is shifted to the blue (Southall and Cogdell, unpublished data). Notice that a very similar assumption has been made by Zigmantas *et al.*^35^ in order to explain the 30 nm blue-shift present in the absorption band of the B820 ring in the LH4 complex. This assumption is supported also by the crystal structure^36^. Therefore the energy of the CT states or polaron pair would be expected to be shifted as well^32^. However, it has been shown that in a nanoscale system, the manifold of CT states and polaron pairs varies, with the presence of many different states that span from the blue to the red side of the excitonic band, meaning that in spite of the shift, both M-LH2 and WT-LH2 may have states in the 850 nm region. A simplified model is shown in the next section to illustrate how the energy manifold of red-lying CT states changes, if the Bchls electronic energy is shifted to the blue (WT-LH2 vs. M-LH2).

Normally only few of these dark states are relevant for the dynamics, and Polivka *et al.*^34^ propose that polaron pairs emitting in the 850–870 nm region may be used for localizing the excitation on special BChls dimers of LH2 that are in close contact with LH1, creating a B850 “red shuttle” site that increases the LH2-LH1 energy transfer efficiency.

According to this model, in WT-LH2 the X state is resonant with the exciton band B850 and therefore because of localization of the energy, it can increase the energy transfer efficiency. However, in M-LH2 the X state is not resonant with the exciton band (B810), thus it acts as an energy trap.

**Figure 4:**
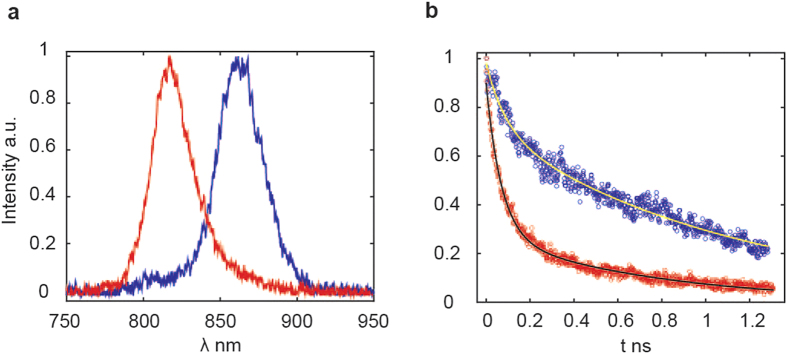
Time resolved fluorescence. (**a**) emission fluorescence spectra after few ps of WT-LH2 exciton band (blue) and M-LH2 exciton band (red). The maxima of emission are at 866 and 816 nm respectively. (**b**) Time resolved traces corresponding to the emission maxima of 866 nm for WT-LH2 (blue) and 816 nm for M-LH2 (red). The traces are fit (solid lines) with mono exponential and bi-exponential decay respectively. The WT-LH2 band shows a life time of about 1.2 ns, whereas the M-LH2 band shows a fast decay of 70 ps and a slow decay of 700 ps.

The relaxation life times of the B810 and B850 bands have been measured from the time-resolved fluorescence traces (Fig. 4b), in order to determine if in M-LH2 the energy is quenched. For these measurements both complexes were excited at 790 nm, and the traces in Fig. 4b correspond to the maximum of the emission, which is 816 and 866 nm for M-LH2 (B810) and WT-LH2 (B850), respectively. With an exponential fit it is possible to calculate the emission life times, which are 70 ps for the B810 band and about 1.2 ns for B850 band (the WT-LH2 relaxation life time is in agreement with previous work^37^). These results show the strong energy quenching in M-LH2, and from the life times ratio it is possible to calculate the probability of quenching which is 90%. Notice that in M-LH2 the trace shows a bi-exponential decay. The slower component is about 700 ps, this is of the same order of that of the B850 band. This shows that in about 30% (ratio between the amplitudes of the exponential decays) of M-LH2, the quenching is significantly weaker.

### Model

**Figure 5:**
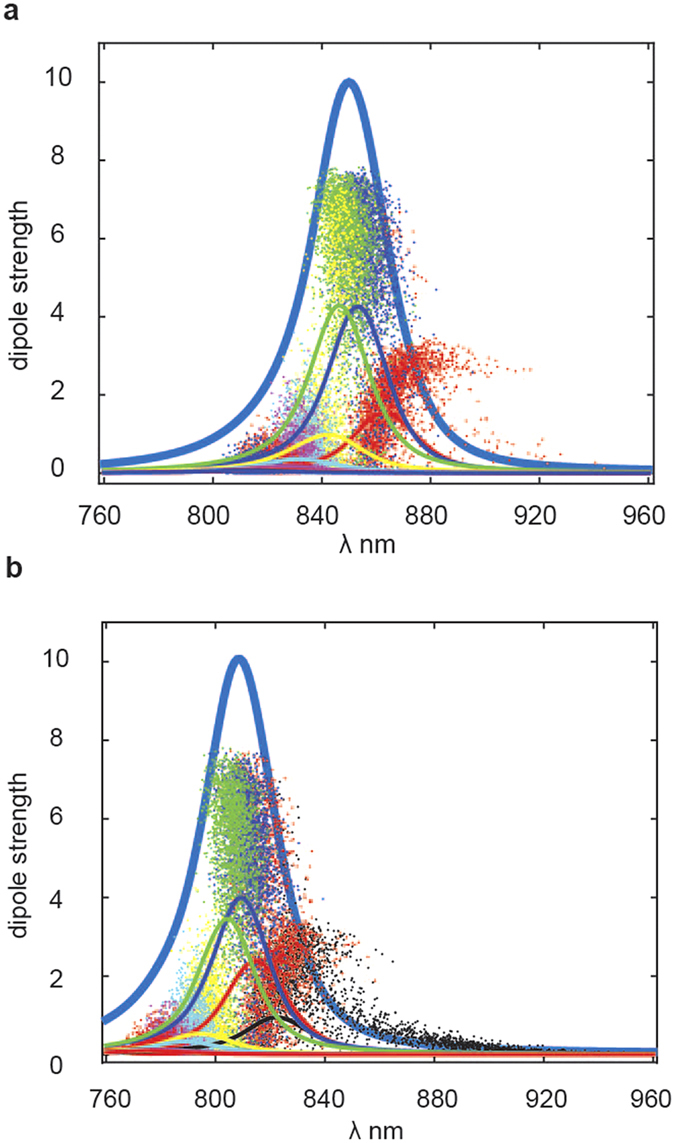
The model. In this simulation, the points corresponds to the dipole strength, in units of the monomeric dipole strength (*y*-axes), whereas lines shows the absorption of the corresponding exciton component. Panel **a** represents the absorption of the B850 band (as in WT-LH2), whereas panel **b** the absorption of the B810 band (as in M-LH2). The superradiant exciton states, corresponding to k = ±1,0 transitions are shown by blue, green, and red, respectively. The localized CT states, which act as energy traps, are shown in black.

In this model it is assumed that the 18 BChls of the dimeric LH2 ring are coupled to a single CT state. We suppose an exciton-type mixing between the excited states and CT. The exciton couplings, site inhomogeneity and exciton-phonon spectral density for BChls are the same as in our previous modelling of LH2^38^, whereas the inhomogeneity value and exciton-phonon couplings for the CT are supposed to be 1.6 times bigger than for excited states^39^. The absorption spectra are calculated using the modified Redfield theory^38^. The results are shown in Fig.5a,b, where the points represent the distribution of the dipole strength, whereas the lines show the absorption of the corresponding exciton component together with the total absorption profile.

In the B850 example, the site energy of the diabatic CT state is the same as for the BChls of the B850 band. Thus the CT states are uniformly mixed with the excited states, producing delocalized eigenstates looking very similar to the usual exciton states of the ring (with most intense k = ±1 transitions and a less intense, but still superradiant lowest level (k = 0), shown by blue, green, and red, respectively). These states have reduced reorganization shifts compared to the localized states, meaning that the resulting energy of the mixed exciton-CT states is in the blue and middle region, around the most intense k = ±1 states, peaking near 850 nm.

In the B810 example we change the site energies of BChls (in order to describe the absorption blue shift of about 40 nm), whereas the energy of the diabatic CT state is assumed to be the same as in the previous example. Thus, in this case the CT states are not so strongly mixed with the exciton states due to the large energy gap. As a consequence the mixed exciton-CT states are more localized and have a bigger reorganization shift compared to the B850 case. Consequently, they are redder than the B810 exciton band. (black dots and line in Fig. 5b). Notice that in Fig. 5b, only the CT states that act as energy traps are represented in black.

We conclude that in WT-LH2 (B850) strong mixing and delocalization keep CT states in the region of the major exciton transitions, whereas in M-LH2 (B810) less mixing produces localized and strongly red shifted CT states with a small amount of exciton character.

### Frequency maps

**Figure 6:**
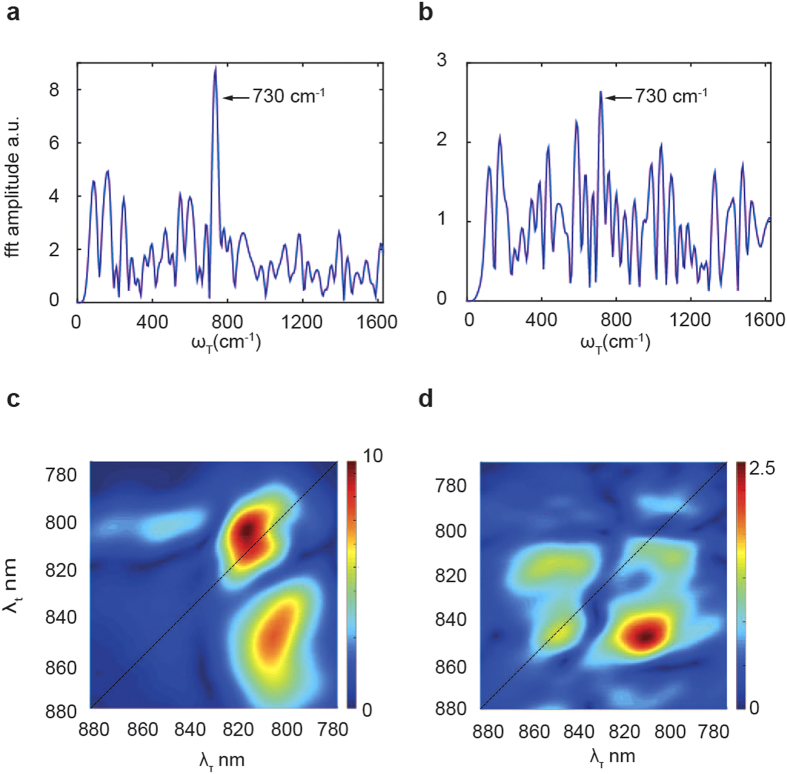
Frequency maps. (**a**) FFT of the (810, 850) nm trace of M-LH2. (**b)** FFT of the (805,850) nm trace of WT-LH2. (**c–d)** frequency maps corresponding to the beating frequency *ω_T_* = 730 cm^−1^ for M-LH2 (**c**) and WT-LH2 (**d**).

Amplitude oscillations (beats), as a function of *T*, are clearly present in the traces of the positive below diagonal peak (810,850) nm for M-LH2 in Fig. 3c, and (805, 850) nm for WT-LH2 Fig. 3d. To show this more clearly the exponential rise was removed from the traces and the residuals were analysed applying a fast Fourier transformation (FFT) algorithm in order to calculate the beating frequencies *ω*_*T*_ (Fig. 6a,b) as reported before^12^,^20^. In both cases (M-LH2 Fig. 6a, WT-LH2 Fig. 6b) a dominant peak appears at *ω*_*T*_ = 730 cm^−1^. This vibrational frequency has been reported before in the BChls dimer B820^20^, which has a structure similar to one of the B850 subunits, and it is resonant with the difference in energy between B800 and B850 states. In the M-LH2 the peak is even more intense, but in this case the energy matches only the difference between the B810 and X states, because the ‘normal’ B850 band is missing.

Furthermore, the amplitude contribution to the beating frequency *ω*_*T*_ = 730 cm^−1^ from each point of the 2D spectra is represented in the 2D frequency maps for M-LH2 in Fig.6c and WT-LH2 in Fig.6d. The main advantage of the 2D frequency map representation is that it provides higher selectivity than 2D spectra, in fact the former show only the beating states^12^. As expected, both frequency maps are dominated by a below diagonal peak. From this peak we can conclude that the CT state of M-LH2 (Fig.6c) is emitting at 855 nm.

## Discussion

The energy transfer dynamics of light harvesting complexes in purple bacteria has been the subject of detailed studies over the past decades. The presence and possible role of CT states and/or polaron pairs has been proposed in different theoretical models in order to explain the ultra-fast dynamics of different photosynthetic complexes^4^,^11^,^14^,^30^,^34^. The CT states have been proposed to be involved in energy transfer, in spite of their small transition dipole moment. In fact in this case the transition dipole moment of the CT states is enhanced because of the resonance between the CT states and the exciton bands in both LH1 and LH2. In particular, evidence exists which indicates CT states are necessary in LH2 for intra-band energy transfer^29^ and for red lying CT states to be required for the formation of polaron pairs that enhance the LH2-LH1 inter-bands energy transfer^34^.

The present study shows direct evidence for the presence of an almost dark state in M-LH2, which, taken together with conclusions of previous work, suggests the presence of red lying CT states or polaron pairs in LH2 antennae. Analysis of the cross-peaks amplitudes of 2DES spectra shows that the population of the CT state or polaron pairs occurs in only 50–65 fs.

An analysis of the WT-LH2 dynamics also shows a signature of the presence of the almost dark state in the 850 nm region, however for WT-LH2 it is not possible to locate the exact position of this state.

The present paper shows the strength of 2DES in revealing dark states and supports the conclusions of many previous studies indicating that the CT states and/or polaron pairs are present in the LH2 antenna, playing a fundamental role in the dynamics. In fact in WT-LH2, the CT states are uniformly mixed with exciton states and they may act as a gateway for energy transfer to LH1-RC. Differently, in the M-LH2 mutant they act as energy quencher since a resonant exciton band is absent. The strong energy quenching is supported by time resolved fluorescence measurements, which show that in M-LH2 about 70% of excitation is quenched, with a life time of about 70 ps. Although the presence of a fast emission component supports the results obtained from the 2DES traces, the life time of 70 ps is much longer than the energy transfer time from B810 to CT states, that was found to be 70 fs. In fact the 70 ps component represents the life time of the dark state.

The fact that optically forbidden states may act as strong energy quenchers is well known since early studies of Porter *et al.*^40^. This is a natural consequence of highly concentrated pigments, which leads to the formation of red lying optically forbidden states, such as charge transfer states, which would quench the excited state, not very desirable for natural photosynthesis. In more recent works^2^, it has been shown how directionality in the flow of energy can avoid the energy quenching and how the heterogeneity of protein conformation can shift the lowest (B850) absorption band in LH2 to the red. This work represents an example of how concentration quenching can occur via CT (or more in general almost-dark) states that are redder than the lowest exciton band, like in M-LH2; and how the problem of quenching is solved in the wild type LH2 (WT-LH2), where a heterogeneous/red-shifted protein conformation is present.

Notice that in spite of all the theoretical models predicting that the CT states originate from the strong coupling between the BChl dimers in the B850 ring, the experimental observation that the energy of such states is unaltered in the mutant, suggests that that perhaps this dark state is associated with the B800 ring. In fact the B800 ring is not as dramatically affected in the mutant. Further theoretical modelling of the 2D spectra is necessary to locate the exact position of the state in WT-LH2 and to decide if the nature of the dark states is a CT state and/or a polaron pairs.

## Methods

### Sample preparation

The LH2 complexes were isolated and purified from wild-type *Rhodopsuedomonas palustris* and the deletion mutant that only contains the *puc*D LH2 genes using the standard protocols described in detail in Brotosudarmo *et al.*^22^. The purified LH2 complexes were used in 20 mM Tris HCl pH 8.0 buffer containing 0.1% lauryldimethylamine-n-oxide (LDAO).

### Spectroscopy

2D electronic spectra were recorded at room temperature with a passively stabilized diffractive optics based set-up, which was described previously in detail^41^. The coherence time (*τ*) is scanned from −70 fs to 120 fs, with a step of 1 fs. The population time (*T*) is scanned from 0 to 1 ps, with a 10 fs step. In addition, spectra at longer *T* (5, 10 and 100 ps) are recorded. The laser system (PHAROS, Light Conversion) repetition rate is set to 1 kHz. The laser pulses are generated using a home-built non-collinear optical parametric amplifier (NOPA). Each pulse is centered at 810 nm with a full width half maximum (FWHM) of 90 nm. The pulse duration is 11 fs, the energy per pulse is 5 nJ and the spot diameter on the sample is about 100 μm.

For the time resolved fluorescence experiments the samples were excited at 795 nm using a pulsed Titanium:Sapphire (Ti:Sa) laser system, consisting of a mode-locked oscillator (Vitesse, Coherent) and a regenerative amplifier (ReGa, Coherent), operated at a repetition rate of 50 kHz, a pulse width of about 200 fs and an energy per pulse of 10 nJ. The emitted signal, polarized at the magic angle, was detected at 90° with respect to the excitation beam by a streak camera device. The instrument response function (IRF) is about 4–5 ps, which is completely limited by the detector response.

## Additional Information

**How to cite this article**: Ferretti, M. *et al.* Dark States in the Light-Harvesting complex 2 Revealed by Two-dimensional Electronic Spectroscopy. *Sci. Rep.* **6**, 20834; doi: 10.1038/srep20834 (2016).

## Figures and Tables

**Figure 1:**
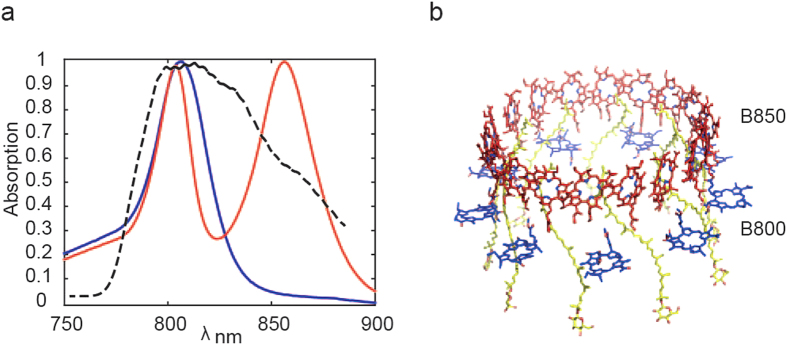
The LH2 antenna. (**a**) Absorption spectra for M-LH2 (blue) and WT-LH2 (red) and laser spectrum profile (dash). (**b**) Crystal structure of LH2 from *Rps. acidophila*.
